# Optimizing Belantamab Mafodotin in Relapsed or Refractory Multiple Myeloma: Impact of Dose Modifications on Adverse Events and Hematologic Response in a Real-World Retrospective Study †

**DOI:** 10.3390/cancers17142398

**Published:** 2025-07-19

**Authors:** Lina Zoe Rüsing, Jakob Schweighofer, Julia Aschauer, Georg Jeryczynski, Lea Vospernik, Heinz Gisslinger, Armin Marcus Bumberger, Julia Cserna, Julia Riedl, Hermine Agis, Maria-Theresa Krauth

**Affiliations:** 1 Department of Medicine I, Medical University Vienna, 1090 Wien, Austria; georg.jeryczynski@meduniwien.ac.at (G.J.); n11922547@students.meduniwien.ac.at (L.V.); heinz.gisslinger@meduniwien.ac.at (H.G.); armin.bumberger@meduniwien.ac.at (A.M.B.); julia.cserna@meduniwien.ac.at (J.C.); julia.riedl@meduniwien.ac.at (J.R.); hermine.agis@meduniwien.ac.at (H.A.); maria.krauth@meduniwien.ac.at (M.-T.K.); 2 Department of Ophthalmology and Optometry, Medical University Vienna, 1090 Wien, Austriajulia.aschauer@meduniwien.ac.at (J.A.)

**Keywords:** multiple myeloma, antibody–drug conjugate, treatment tolerability

## Abstract

**Background**: Belantamab mafodotin (belamaf) is a BCMA-targeting antibody–drug conjugate used in triple-class refractory multiple myeloma. Despite its efficacy, keratopathy remains a significant dose-limiting toxicity. Following its withdrawal from the U.S. market in 2022, its use in Austria is limited to clinical trials or compassionate use. **Methods**: In this real-world, retrospective study, we analyzed 36 relapsed/refractory, BCMA-naïve multiple myeloma patients treated at the University Hospital of Vienna (January 2020–June 2024); 42% received a reduced dose (1.9 mg/kg) throughout all treatment cycles. The primary objective was to assess adverse events, particularly keratopathy, and the impact of dose modifications on toxicity and efficacy. **Results**: The overall response rate was 64%, with responders having significantly fewer prior therapy lines (median 3 vs. 4.5, *p* = 0.015). Median PFS was 7.3 months, significantly longer in responders (11.1 vs. 1.6 months, *p* < 0.0001); median OS was 20.1 months, also longer in responders (not reached vs. 18 months, *p* = 0.031). Keratopathy occurred in 75% of patients; 33% experienced grade 3–4 events. Dose reduction significantly decreased grade 3–4 keratopathy (7% vs. 52%, *p* = 0.004) and thrombocytopenia (33% vs. 67%, *p* = 0.048) without compromising efficacy. **Conclusions**: Belamaf dose reductions improved tolerability without loss of efficacy, supporting reduced dosing in practice.

## Simple Summary

Belantamab mafodotin (belamaf) is an antibody–drug conjugate used to treat patients with relapsed or refractory multiple myeloma who have limited remaining treatment options. Its clinical use is often limited by ocular toxicity, particularly keratopathy. In this real-world, retrospective study, we evaluated 36 patients treated at the University Hospital of Vienna to assess the safety and efficacy of belamaf, with a focus on the impact of dose modifications. Notably, 42% of patients received a reduced dose throughout all treatment cycles. We found that dose reductions significantly decreased the incidence of severe ocular and hematologic toxicities without compromising treatment outcomes. Real-world data from cohorts like ours may help clinicians to overcome the fear of severe ocular toxicity, which is manageable and even reversible when performing dose adjustments.

## 1. Introduction

Multiple myeloma (MM) is still an incurable disease with a median survival ranging from 2 to 10 years [1]. Although the incidence of MM increases, clinical outcome steadily improves, due to significantly prolonged overall survival (OS) achieved with new therapeutic agents [2,3,4]. Immunomodulatory drugs (IMiDs), proteasome inhibitors (PIs), and anti-CD38-monoclonal antibodies (MoAbs) are the backbone of modern multiple myeloma therapies [3,5]. Quadruplet therapies incorporating all these agents have been approved recently for first-line therapy [6]. However, the disease mostly becomes refractory (RRMM) to prior therapies, and the prognosis of triple-class refractory patients is extremely poor (median OS 12 months) [3,7,8,9].

Belantamab Mafodotin (Belamaf) is a first in class antibody–drug conjugate (ADC), targeting B-cell maturation antigen (BCMA), initially approved for patients with RRMM, who have received at least four prior therapies. It has been withdrawn from the market since the pivotal DREAMM-3 study failed to reach the clinical endpoint [10]. However, promising DREAMM−7 and DREAMM-8 trials, as well as the ALGONQUIN trial, make the re-approval likely [11,12,13]. Belamaf is composed of a humanized immunoglobulin G1 anti-BCMA monoclonal antibody, conjugated to a cytotoxic payload, the microtubule-disrupting agent monomethyl auristatin F (MMAF) [14]. After binding to BCMA, the mAb drug complex is internalized, allowing MMAF to induce apoptosis [15]. The Phase 1 Trial, DREAMM-1 [16,17], analyzed safety and clinical efficacy of belamaf in 38 patients. In the DREAMM-2 trial [14,18], a randomized two-armed phase II study, patients were randomized to receive either 2.5 mg/kg (95 patients, 32% ORR) or 3.4 mg/kg (35% ORR). In both studies, corneal events, thrombocytopenia and anemia were the most common AEs reported [14,18].

Keratopathy, especially corneal microcyst-like epithelial changes (MECs), are frequently associated with antibody–drug conjugates (ADCs) containing MMAF. However, the pathogenesis is unknown [19]. MECs can result in a variety of symptoms ranging from mild to moderate levels of discomfort and irritation, such as blurred vision, foreign body sensation, photophobia, and dry eyes, to severe, vision-threatening events [19,20,21]. Interestingly, upon ophthalmologic examination, corneal changes are not always accompanied by patient symptoms or changes in best-corrected visual acuity (BCVA). Since no linear relationship was observed between the severity of keratopathy (MECs) and changes in BCVA in patients receiving belamaf, it is recommended that corneal events should be graded using the Keratopathy and Visual Acuity (KVA) scale based on the worst finding of either keratopathy (MECs) on the ophthalmologic eye examination or BCVA assessment [21].

The primary objective of this study is to evaluate the incidence of AEs, specifically keratopathy/MECs, in patients treated with belamaf and to assess whether dose-adjustments have an impact on ocular side effects. In addition, we aim to investigate if dose modifications have an impact on clinical efficacy (ORR, PFS, and OS). Furthermore, we tried to elaborate on potential genetic, clinical, or laboratory markers that could predict response or non-responsiveness to belamaf therapy. Secondary objectives include determining the time to the first and best hematological response and comparing PFS and OS between responders and non-responders.

## 2. Materials and Methods

This retrospective, single-center cohort study evaluated 36 multiple myeloma patients treated with belamaf at the University Hospital Vienna between 1 January 2020 and 6 January 2024. All patients were treated in an outpatient setting and had at least two months of follow-up. No interventions were performed; data were extracted from electronic medical records. The study was approved by the ethics committee of the Medical University of Vienna (EK Nr: 1801/2024) and adhered to the Declaration of Helsinki. Belamaf was administered every 21 days, with dosing delayed in cases of AEs until toxicity resolved to grade 1 or lower. Preliminary results were presented as an abstract at the EHA 2025 Congress, Milan, Italy [22].

Ophthalmologic examinations were scheduled prior to the first dose and then before each of the subsequent doses/cycles, even in the absence of symptoms, and were performed by a certified ophthalmologist at the Department of Ophthalmology and Optometry at the Medical University of Vienna. Keratopathy was graded using the Keratopathy and Visual Acuity scale (KVA-Scale). Besides evaluation based on the KVA scale, all patients underwent slit-lamp photography, corneal esthesiometry, corneal epithelial thickness mapping using anterior segment optical coherence tomography, and corneal confocal microscopy to characterize corneal epithelial and sub-epithelial changes in an en-face view with micrometer resolution. As part of prophylactic measures to reduce ocular toxicity, all patients were instructed to use preservative-free lubricating eye drops (artificial tears) at least four times daily, starting before the first infusion and continuing throughout treatment. Given the absence of clinical data and official recommendations, we opted not to use topical corticosteroid eye drops as a preventive measure. In cases of moderate keratopathy (KVA Grade 2–3), belamaf dose was reduced to 1.9 mg/kg and treatment intervals were extended. Due to treatment-limiting keratopathy observed at our center and similar safety concerns reported by other institutions, some patients were started on a reduced dose of 1.9 mg/kg for all treatment cycles. Patients who received all cycles at 1.9 mg/kg were classified as the reduced-dose cohort, whereas those who received at least one cycle at 2.5 mg/kg were classified as the full-dose cohort.

### 2.1. Study Endpoints

To evaluate the impact of dose reduction, we conducted a retrospective comparison of patients receiving reduced dose versus full dose of belamaf, assessing keratopathy incidence, PFS, and OS. Hematologic response was evaluated using updated IMWG criteria [23]. We merged stringent CR and CR, defined as negative serum and urine immuno-fixation, because not all patients underwent bone marrow biopsy. Therapy lines were defined per Rajkumar et al. [24]. The National Cancer Institute Common Criteria for AEs version 5.0 was utilized to define AEs. High-risk cytogenetics were defined as the presence of any of the following aberrations: t(4;14), t(14;16), del(17p), or 1q21 gain or amplification.

### 2.2. Statistical Analysis

For nominal variables, the absolute and relative frequencies are listed in tabular form. For metric variables, the median and ranges are displayed in tabular form. Continuous data was compared using the nonparametric Mann–Whitney U test or *t*-Test as appropriate, and categorical data was compared using the χ^2^ or Fisher’s exact test, as appropriate. Survival probabilities were estimated by the Kaplan–Meier method. A significance level of *p* = 0.05 was used for all statistical tests. Given the exploratory design, small cohort size, and possible limited statistical power, *p*-values were not adjusted for multiple comparisons and should be interpreted descriptively and cautiously, as hypothesis-generating rather than confirmatory.

PFS and OS were compared between responders and non-responders, defined at a 3-month landmark. PFS was analyzed using a Cox proportional hazards model with treatment response as a time-dependent covariate, up to 3 months. This approach was selected to avoid excluding patients who progressed before response assessment and to minimize immortal time bias. Patients were considered non-responders until a response (≥PR) was documented, and responders thereafter. OS was assessed using a landmark analysis at 3 months. Only patients alive at the landmark timepoint were included. Response status at 3 months was used to classify patients as responders or non-responders. OS was then analyzed from the landmark onward using Kaplan–Meier estimates, with group comparisons performed using the log-rank test and Cox proportional hazards modeling. All statistical analyses were performed using SPSS Statistics, version 29.0.2.0 (IBM Corp., Armonk, NY, USA), and GraphPad Prism, version 10.3.1 (GraphPad Software, San Diego, CA, USA).

## 3. Results

### 3.1. Patients, Disease and Treatment Characteristics

A total of 36 patients were included in this study, with a median age of 66 years (range: 54–87); 61% were female and 39% male. Paramedullary disease (PMD) was present in 50% of patients, primarily bone-associated (*n* = 11), while 20% (*n* = 7) had soft tissue extramedullary disease (EMD). Osteolytic lesions were observed in 89% of cases. Patients had received a median of four prior lines of therapy (range: 2–9). All had been treated with anti-CD38 monoclonal antibodies, proteasome inhibitors (PIs), and immunomodulatory drugs (IMiDs); none had prior exposure to BCMA-targeted therapies. Autologous stem cell transplantation (ASCT) had been performed in 64% (*n* = 23). A complete response (CR) to any previous treatment line was reached by 67% (*n* = 24). ORR to the most recent therapy line was 67%. Median progression-free survival (PFS) to the last treatment was 11.5 months (range: 0.5–36.9), and the median therapy-free interval before receiving belamaf was 42 days (range: 2–181) (Table 1).

The median number of belamaf cycles administered was five (range: 1–27). Approximately one third (31%) of our patients received belamaf as a combinational therapy, including combinations with proteasome inhibitors (PIs), immunomodulatory drugs (IMiDs), anti-CD38 monoclonal antibodies, and chemotherapeutic agents (Table 1). Treatment intervals were extended beyond four weeks in approximately 40% of patients at some point during therapy. The primary reasons for prolonging treatment intervals were keratopathy management and prevention. The median cycle duration was 31 days (range, 21–80 days) based on the overall treatment period, as cycle lengths varied during the course of therapy.

Dose reduction to 1.9 mg/kg for at least one cycle occurred in 72% of patients, also predominantly as a strategy to mitigate keratopathy. Notably, 42% of patients received a reduced belamaf dose from the initial start of therapy and maintained this reduced dose for all subsequent cycles.

### 3.2. Response to Belamaf

**Figure 1:**
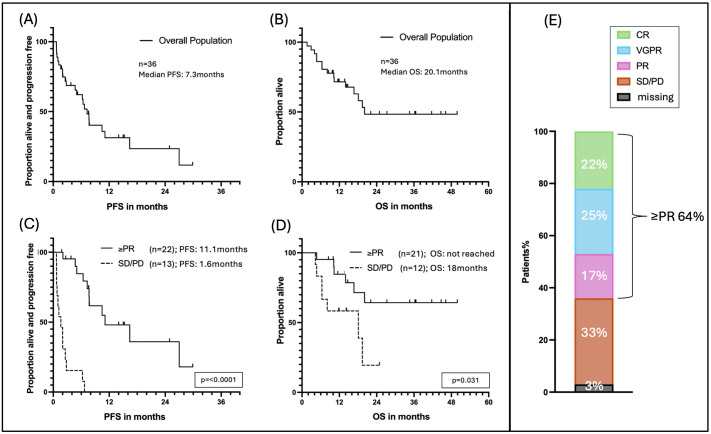
PFS and OS of responders and non-responders to belamaf—3-month landmark: (**A**) PFS complete cohort; (**B**) OS complete cohort; (**C**) Kaplan–Meier analysis of progression-free survival (PFS) comparing responders and non-responders, with treatment response (≥PR) modeled as a time-dependent covariate starting at 3 months. All patients with evaluable response data were included (*n* = 35). (**D**) Kaplan–Meier analysis of overall survival (OS) from a 3-month landmark, including only patients alive at the landmark timepoint and stratified by response status at that time. Of 36 patients, 3 were excluded from the responder vs. non-responder analyses: 2 patients died prior to the 3-month landmark and were excluded from the OS analysis, while 1 patient had no evaluable response data and was excluded from both analyses. An additional patient who achieved a partial response only after day 102 was classified as a non-responder in both analyses. (**E**) Overall response rate (ORR) complete cohort.

The overall response rate was 64%. Of these responders, eight patients (22%) achieved a CR, nine patients (25%) reached a VGPR, and six patients (17%) had a PR. Twelve patients (33%) did not respond to belamaf. The median time to first observed response (≥PR) was 28 days with a range of 8 to 102 days (Table 2, Figure 1E). Patients with a deeper belamaf response (≥VGPR) already showed earlier first response than patients with PR (median 23 days vs. median 71.5 days; *p* = 0.009) (Figure S1). The median time to the best observed response, regardless of the type of response (CR/VGPR/PR), was 72 days, with a broad range of 14 to 347 days. Twelve responders who initially had ≥PR subsequently deepened their response. Median time from first to best observed response was 60 days (range: 9–327 days) (Table 2). A total of 32 patients (89%) discontinued belamaf therapy due to different reasons: 58% of patients (*n* = 21) discontinued belamaf due to disease progression (PD) or insufficient response. Eleven patients (31%) discontinued therapy even though the response was sufficient. Eight patients (22%) discontinued because of keratopathy, one patient (3%) discontinued due to bicytopenia, predominantly severe thrombocytopenia, and two patients (6%) stopped treatment for reasons unrelated to either belamaf or disease progression. At the time of data cut-off, four patients (11%) were still receiving belamaf (Table 1).

Median progression-free survival (PFS) for the entire cohort was 7.3 months, and median overall survival (OS) was 20.1 months (Figure 1A,B). To assess the clinical impact of treatment response, PFS and OS were compared between responders (≥PR) and non-responders, using a 3-month landmark. Responders demonstrated a significantly longer median PFS of 11.1 months compared to 1.6 months in non-responders (*p* < 0.0001) (Figure 1C). Similarly, landmark OS was significantly longer in responders, with median OS not reached, compared to 18 months in non-responders (*p* = 0.0045) (Figure 1D).

### 3.3. Predictive Markers for Belamaf Response

**Figure 2:**
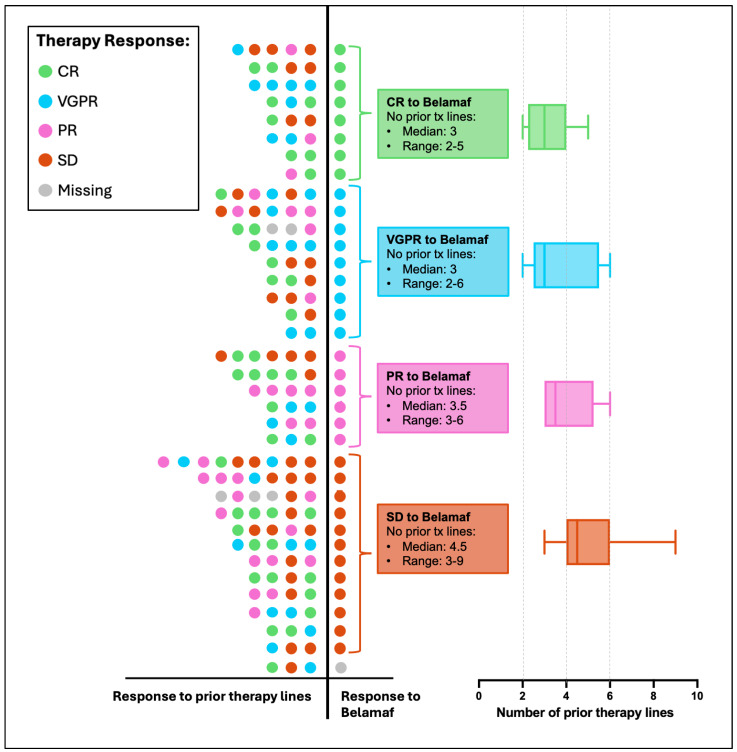
Response to prior therapy lines and response to Belamaf. The 36 belamaf patients were classified into four groups based on their best observed response to belamaf. Each row of dots represents the therapeutic history of an individual patient, with each dot symbolizing one therapy line. The color of the dot indicates the best observed response to that particular therapy line. Dots positioned on the left side of the Y-axis correspond to prior therapy lines, while the dot on the right side reflects the response to belamaf. Patients are grouped based on their response to belamaf, with groups arranged from top to bottom in the order of response: complete response (CR), very good partial response (VGPR), partial response (PR), stable disease (SD), and missing.

To identify predictive markers of response to belamaf, we analyzed baseline clinical and laboratory parameters (Table S1). The only variable that showed a statistically significant association with response to belamaf was the number of prior therapy lines. Belamaf responders (≥PR) had a significantly lower number of prior therapy lines (median 3) compared to non-responders (median 4.5) (*p* = 0.015) (Figure 2, Table S1).

### 3.4. Adverse Events

Within the entire cohort, 75% of patients (*n* = 27) developed keratopathy of any grade during treatment with belamaf. Notably, 33% of patients experienced severe keratopathy of grade 3 or higher, with 14% developing grade 3 and 19% developing grade 4 keratopathy.

In eight patients (22%), this led to discontinuation of belamaf treatment despite having a response. Median time to first documented ocular toxicity was 41 days.

After belamaf discontinuation, subjective visual improvement was described by all patients. Among those with ophthalmologic follow-up (*n* = 14), seven patients achieved complete resolution of keratopathy, and seven patients showed improvement of keratopathy. Nine patients did not attend further ophthalmologic evaluations, and four patients have ongoing belamaf therapy. The median time to improvement was 75.5 days (range: 36–380 days) (Table 3, Figure S2). Thrombocytopenia was observed in 53% of patients (≥Grade 3: 22%) with 22%). In one patient (3%), belamaf therapy was discontinued solely due to thrombocytopenia. Infections occurred in 22%, most common were respiratory tract infections (*n* = 6). Two patients experienced liver toxicity (grade 2), primarily cholestasis. One patient underwent liver biopsy that showed lobular and portal inflammation. First increase in cholestatic enzymes was observed after 15 months in one patient and after 10 months in the other patient. Two patients experienced chronic kidney disease grade 3. One patient underwent a kidney biopsy, which revealed mild to intermediate arteriosclerosis, fibrosis, and tubular atrophy. Electron microscopy showed mild endothelialopathy and no indication of involvement related to myeloma. Hospitalization was required in 22% of patients (*n* = 8), with infections being the primary reason in five of these cases.

### 3.5. Effect of Dose Reduction

**Figure 3:**
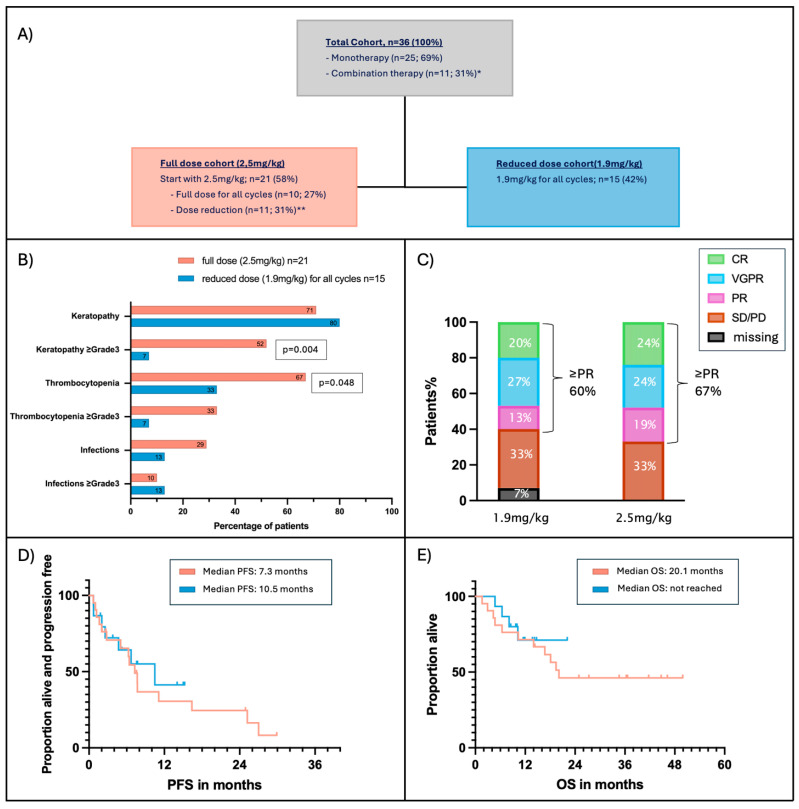
Influence of belamaf dose reduction on incidence of adverse events and efficacy. (**A**) Patients were divided into two groups: 21 patients who started belamaf therapy with the standard dose of 2.5 mg/kg and 15 patients who received belamaf in reduced dose (1.9 mg/kg) for all cycles. (**B**) Influence of belamaf dose on the incidence of adverse events; (**C**) influence of belamaf dose on ORR; (**D**) influence of belamaf dose on PFS; (**E**) influence of belamaf dose on OS. * Various combinations with PIs (Carfilzomib *n* = 2; Bortezomib *n* = 5; Ixazomib *n* = 1); IMiDs (Pomalidomide *n* = 1; Lenalidomide *n* = 1); CTX (Bendamustine *n* = 1; Cyclophosphamide *n* = 1); alternating with D-PACE (*n* = 1); Daratumumab *n* = 1. ** Reason for dose reduction: keratopathy: *n* = 6; keratopathy prophylaxis: *n* = 4; thrombocytopenia: *n* = 1.

Forty-two percent of patients received a reduced belamaf dose from the start of therapy and maintained this reduced dose throughout all subsequent cycles (Figure 3A). When comparing the reduced-dose cohort with the full-dose cohort, the median time since diagnosis was similar (4.3 vs. 4.4 years), and the number of prior therapy lines was comparable (three vs. four, respectively). However, progression-free survival (PFS) on the last prior therapy line was significantly longer in the reduced-dose cohort (17 months vs. 4 months; *p* = 0.021), and combination therapies were more frequently used in the full-dose cohort (43% vs. 13%; *p* = 0.077) (Table S2). The median belamaf dosing interval was similar in both groups (33 vs. 30 days). Patients in the reduced-dose cohort received a median of four belamaf cycles, compared to five cycles in the full-dose cohort.

As described previously, within the full-dose cohort, approximately half of the patients eventually had their dose reduced to 1.9 mg/kg during the course of treatment. When comparing patients who maintained the full dose throughout all cycles to those who received a dose reduction at some point, we found that patients who were reduced to 1.9 mg/kg received significantly more belamaf therapy cycles (median 6.5 vs. 4 cycles; *p* = 0.016) (Figure S3).

Patients who received reduced-dose belamaf throughout all treatment cycles demonstrated significantly lower rates of grade 3–4 keratopathy compared to those who received the full dose (7% vs. 52%; *p* = 0.004), while the overall incidence of keratopathy of any grade was similar between groups (71% vs. 80%) (Figure 3B). Moreover, the median time to keratopathy improvement after treatment discontinuation was significantly shorter in the reduced-dose group (62 vs. 137.5 days; *p* = 0.045) (Figure S2).

Similarly, the incidence of thrombocytopenia of any grade was significantly lower in the reduced-dose cohort compared to the full-dose group (33% vs. 67%; *p* = 0.048). While grade 3–4 thrombocytopenia was also less frequent in the reduced-dose group (7% vs. 33%), this difference did not reach statistical significance (*p* = 0.064). Infections (any grade) were more common in the full dose cohort (29%, *n* = 6) than in the reduced dose cohort (13%, *n* = 2), but not statistically significant (*p* = 0.25) (Figure 3B, Table S2).

Reducing the dose of belamaf did not affect PFS, OS, or ORR. A hematologic response was attained by 67% of patients (*n* = 4) in the full-dose cohort and by 60% (*n* = 9) in the 1.9 mg/kg cohort (Figure 3C–E).

## 4. Discussion

This study provides a comprehensive evaluation of the clinical efficacy and safety of belamaf in a cohort of heavily pretreated patients with RRMM. Our findings align with and build upon previously published data, offering insights into real-world management of belamaf, particularly focusing on the impact of dose adjustments on clinical efficacy and safety (Table 4).

The observed ORR of 64% in this cohort is consistent with previously reported results from clinical trials and retrospective studies, which ranged from 29% to 67% [10,13,14,16,25,26,27,28,29]. Interpreting published data (Table 4), it seems that fewer prior lines of therapy correlate with improved ORR: Abeykoon et al. (2022) reported an ORR of 29% in a heavily pretreated population (median eight prior lines), whereas in the DREAMM-6 trial 67% ORR was seen (median three prior lines) [12,27,29]. This aligns with our findings, where responders had significantly fewer prior therapies than non-responders (median 3 vs. 4.5, *p* = 0.015). In our cohort, most treatment responders exhibited an early hematologic response within one month of therapy (median 28 days), consistent with findings from Shragai et al. (median 23 days). Notably, patients who ultimately achieved a CR or VGPR demonstrated a significantly earlier initial response compared to those who attained only PR (median 23 vs. 71.5 days). This information could be valuable in clinical decision-making, helping to avoid switching therapy too early. PFS (7.3 months) and OS (20.1 months) are also within the reported range (Table 4).

The incidence of ocular toxicity was higher in our study (≥grade 1: 75%; ≥grade 3: 33%) compared to others (range 12–78%), although nearly half of the patients received a reduced dose of belamaf (1.9 mg/kg bodyweight) [10,13,14,16,25,26,27,28,29]. These results may be explained by the extensive ophthalmologic work-up during the treatment cycles and the high frequency of specialized ophthalmologic follow-up. In contrast to other studies, ophthalmologic evaluation was performed prior to each treatment cycle in our patients [10,25,26]. The median time to keratopathy onset was 41 days (range: 18–96), consistent with previously reported data, including DREAMM-2 (36 days), Hultcrantz et al. (39 days), and Abeykoon et al. (42 days) [14,25,27]. These findings highlight a predictable timeframe for keratopathy development, enabling clinicians to implement timely monitoring and intervention strategies. In our cohort, keratopathy was the sole reason for treatment discontinuation in 22% of patients. In DREAMM-7 and DREAMM-8, keratopathy was also commonly observed but effectively managed through dose modifications and resolved within 9–12 weeks, consistent with our observations (median 10 weeks) [12].

In our cohort, 42% (*n* = 15) of patients received all treatment cycles at a reduced dose of 1.9 mg/kg. This was associated with a significantly lower incidence of grade 3–4 keratopathy, as well as a shorter median time to keratopathy resolution after treatment discontinuation (62 vs. 137.5 days; *p* = 0.045).

Additionally, the incidence of thrombocytopenia (all grades) was significantly lower in the reduced-dose group, without any negative impact on PFS, OS, or ORR. These findings align with data from DREAMM-6, ALGONQUIN, DREAMM-7, and DREAMM-8 trials, which support the use of dose modifications to improve tolerability while preserving clinical efficacy (Table 4) [11,12,13,29]. In the phase I/II study by Terpos et al., belamaf-Rd (belamaf, lenalidomide, dexamethasone) was evaluated in transplant-ineligible newly diagnosed patients. High response rates (VGPR or better: 83.3%) were observed across all dose levels, with less keratopathy in the 1.9 and 1.4 mg/kg groups. These findings support our results, suggesting that further dose reduction can maintain efficacy while improving tolerability [30].

Differences in treatment regimens across studies complicate direct comparisons. In our cohort, 31% of patients received various combination regimens. Due to this heterogeneity and the size of our cohort, we did not perform a comparative analysis of different combinations. Notably, as detailed in Supplementary Table S2, only two patients in the low-dose cohort received combination therapy, compared to 9 out of 21 patients in the standard-dose group. This indicates that the observed comparable efficacy of the lower dose cannot only be attributed to a higher frequency of combination therapy in that cohort. While combination therapies are not generally associated with increased ocular toxicity, they may contribute to an elevated risk of hematological adverse events. This potential confounding factor should be considered when interpreting toxicity profiles. However, DREAMM-7 and DREAMM-8 trials showed that belamaf can be administered relatively safely when given dose-adjusted in combination with other potent drugs, thereby preventing severe ocular toxicity but increasing clinical efficacy at the same time [11,12].

Regarding its place in the treatment landscape of multiple myeloma, belamaf has demonstrated meaningful activity in heavily pretreated patients and may continue to play an important role as a later-line option, particularly in triple-class–refractory or penta-refractory disease. However, further prospective clinical trials are needed to address important open questions regarding the optimal use of and duration of belamaf therapy. Specifically, sufficient data on ocular long-term safety have to be generated, since keratopathy is the most important and almost the only clinically relevant adverse event of belamaf. Our real-world data add to the understanding of how to decrease ocular side effects (via dose modification) while maintaining efficacy of belamaf and therefore positively impacting patients’ daily lives.

However, other potentially meaningful long-term adverse events also need to be analyzed in more detail, e.g., secondary primary malignancies, since these play an important role for patients with improved clinical survival. The ideal dose and schedule to maximize efficacy while minimizing toxicity need to be elaborated extensively to further improve the quality of life of myeloma patients. This holds true when belamaf is used as monotherapy, but is even more important when used in combination with other drugs.

Emerging data from DREAMM-7 (combination with bortezomib and dexamethasone, BelaVd) and DREAMM-8 (combination with pomalidomide and dexamethasone, BelaPomd) studies suggest that belamaf, when given in combination with standard backbones and with dose modifications, has excellent efficacy with beneficial clinical outcome. Based on recently published results of DREAMM-7 and DREAMM-8, rapid approval by the authorities is expected.

Moreover, due to impressive efficacy in patients earlier in the course of their disease, belamaf combinations (BelaVd, BelaPomd) have already found their way into international guidelines, as has been shown in the recently published EHA/EMN treatment guidelines for multiple myeloma [31]. Interestingly, belamaf combos are listed head-to-head with cilta-cel in the setting of first relapse which reflects their potency to be direct competitors to CAR T-cell therapies, which are currently the most praised therapeutic strategies in myeloma treatment.

We are aware that the retrospective nature of this study may include potential biases in data collection, inconsistencies in documentation, and variability in treatment approaches across patients. There were also imbalances between dose groups. Notably, a higher proportion of patients in the standard-dose group received combination therapies (9/21) compared to the low-dose group (2/15), which may confound toxicity comparisons despite the lack of observed differences in efficacy. Furthermore, although baseline characteristics such as age, sex, number of prior lines, and time since diagnosis were similar in groups (Table S2), patients in the low-dose group had significantly shorter PFS on their most recent prior treatment. This may indicate a selection bias, in which patients perceived to be at higher clinical risk were more likely to receive the full 2.5 mg/kg dose. A major limitation of our study is the relatively small cohort size (*n* = 36), which restricts the statistical power, especially for subgroup analyses (e.g., by dose and combination therapy). While our findings provide valuable real-world insights, they should be interpreted as exploratory and hypothesis-generating rather than definitive. Larger, prospective studies are needed to validate these results and better define the clinical utility of belamaf dose adjustments.

## 5. Conclusions

Our study provides valuable insights into the real-world use of belamaf in a heavily pretreated population of patients with relapsed and RRMM. The observed overall response rate is encouraging and underscores the potential efficacy of belamaf in this challenging clinical setting. Notably, our findings are consistent with results from large phase three clinical trials, demonstrating that the efficacy observed in controlled studies translates into real-world practice. However, the high incidence of keratopathy highlights the importance of proactive side effect management, including dose modifications, close monitoring, and close collaboration with ophthalmologists to improve tolerability. In our cohort, dose reductions effectively reduced ocular and hematologic toxicities without compromising clinical efficacy in terms of response rates and progression-free survival.

All in all, our findings add to the current understanding of how to administer belamaf safely in RRMM. This is of particular importance since newer trials show a dramatic increase in the clinical efficacy when combining belamaf with IMIDs or PIs, which will lead to a re-approval of belamaf in the near future. Therefore, data from real-world studies like our cohort may help clinicians to overcome the fear of severe ocular toxicity, which has been shown to be manageable and even reversible when performing dose adjustments.

## Figures and Tables

**Table 1:** Baseline patient, disease, and treatment characteristics of the total cohort.

Baseline Characteristic		Total Cohort *n* = 36 (100%)
Patient	Age: Median (range)	66 (54–87)
	**Sex: No (%)**	
	Women	22 (61%)
	Men	14 (39%)
	Median time (in years) since diagnosis: Median (range)	4.4 (1.2–21.7)
Disease	**ISS stage at screening: No (%)**	
	I	10 (28%)
	II	12 (33%)
	III	12 (33%)
	Missing	2 (6%)
	Multiple Myeloma type: No (%) *	
	Intact (Ig G/A/M/D)	22 (61%)
	FLC	13 (36%)
	Asecretory (no Paraprotein kown)	1 (3%)
	**High risk Cytogenetic markers: No (%)**	
	High risk	11 (31%)
	del(l17p)	5 (14%)
	t(4;14)	3 (8%)
	t(14;16)	3 (8%)
	1q21+	5 (14%)
	Standard risk	9 (25%)
	Missing	16 (44%)
	Soft tissue extramedullary disease: No (%)	7 (19%)
	Bone-related paramedullary disease: No (%)	11 (31%)
	Osteolytic lesions: No (%)	32 (89%)
Prior therapies	No of prior therapy lines: Median (range)	4 (2–9)
	**Best prior observed response to any line: No (%)**	
	Complete response (CR)	24 (67%)
	Very good partial response (≥VGPR)	32 (89%)
	Partial response (≥PR)	36 (100%)
	**Previous PIs: No (%)**	36 (100%)
	*n* = 1	4 (11%)
	*n* = 2	28 (78%)
	*n* = 3	4 (11%)
	**Previous Imids: No (%)**	36 (100%)
	*n* = 1	9 (25%)
	*n* = 2	20 (56%)
	*n* = 3	7 (19%)
	Previous CD38 **: No (%)	36 (100%)
	Prior PACE: No (%) ***	6 (17%)
	Prior Transplant: No (%) ****	23 (64%)
	Mono	12 (33%)
	Tandem	3 (8%)
	2× (Second salvage ASCT)	7 (19%)
	Allo (1× autologous; 1× allogenic)	1 (3%)
	**ORR to most recent tx line: No (%)**	24 (67%)
	Complete response (CR)	8 (22%)
	Very good partial response (VGPR)	8 (22%)
	Partial response (PR)	8 (22%)
	Stable disease/Progressive disease (SD/PD)	12 (33%)
	PFS to last tx line in months: Median (range)	11.5 (0.5–36.9)
	Therapy free interval prior belamaf in days: median (range)	42 (2–181)
Belamaf	**Therapy regimen used: No (%)**	
	Belamaf Mono	25 (69%)
	Belamaf in Combination with other therapies *****	11 (31%)
	Cycles of belamaf: Median (range)	5 (1–27)
	Treatment holiday (one dose only) >21 days: No (%)	6 (17%)
	**Therapy interval: No (%)**	
	3 weeks	22 (61%)
	≥4 weeks ******	14 (39%)
	Dosing interval in days: Median (range)	31 (21–80)
	Percentage of cycles with prolonged tx interval: median (range)	79% (20–100)
	**Reason for modified therapy intervals: No (%)**	
	Keratopathy	9 (25%)
	Keratopathy prophylaxis	3 (8%)
	Thrombocytopenia	1 (3%)
	**Treatment dose: No (%)**	
	2.5 mg/kg start dosis and for all cycles	21 (58%)
	2.5 mg/kg start dosis, reduction to 1.9 mg/kg	11 (31%)
	1.9 mg/kg (for all cycles)	15 (42%)
	Percentage of therapy cycles in reduced treatment dose:	
	Median (range)	100 (3.7–100)
	**Reason for dose reduction: No (%)**	
	Keratopathy	6 (17%)
	Keratopathy prophylaxis	19 (53%)
	Thrombocytopenia	1 (3%)

* IgG Kappa *n* = 10; IgG Lambda *n* = 7; IgA Kappa *n* = 4; IgA Lambda *n* = 2; Kappa light chain *n* = 4; Lambda light chain *n* = 8; asecretory *n* = 1; ** All patients received Daratumumab prior to belamaf, One patients also received Isatuximab prior to Blernep, *** PACE is a combinational chemotherapy regimen consisting of: Cisplatin, etoposide, doxorubicin, cyclophosphamide; **** ASCT Mono *n* = 12; ASCT Tandem *n* = 8; 2× (second salvage ASCT) *n* = 7; Allo (1× autologous; 1× allogenic) *n* = 1; ***** Different combinations with PIs (Carfilzomib = 2; Bortezomib *n* = 5; Ixazomib *n* = 1; Imids (Imnovid *n* = 1; Lenalidomid *n* = 1); Ctx (Bendamustin *n* = 1; Cyclophosphamid *n* = 1); Alternating with D-PACE (*n* = 1); Daratumumab *n* = 1; ****** *n* = 10 with 4–6 week intervals; *n* = 4 with ≥6 weeks interval; 2 patients further switched to an even longer therapy interval 2–3 months “maintenance therapy”.

**Table 2:** Response to belamaf.

Characteristic:	Total Cohort *n* = 36 (100%)
**Overall response rate (≥PR): No (%)**	**23 (64%)**
Complete response (CR)	8 (22%) *
Very good partial response (VGPR)	9 (25%)
Partial response (PR)	6 (17%)
Stable disease/Progressive disease (SD/PD)	12 (33%)
Response missing **	1 (3%)
**Time to first observed response in days: Median (range)**	**28 (8–102)**
Complete response (CR)	29 (8–66)
Very good partial response (VGPR)	20 (12–86)
Partial response (PR)	71.5 (18–102)
**Time to best observed response in days: Median (range)**	**72 (14–347)**
Complete response (CR)	66 (14–347)
Very good partial response (VGPR)	84 (46–218)
Partial response (PR)	72 (27–102)
Time from first to best observed response: Median (range)	60 (9–327)
Primary Reason for Termination of belamaf: No (%) ***	
PD	21 (58%)
Keratopathy	8 (22%)
Thrombocytopenia	1 (3%)
Reason unrelated to belamaf or PD ****	2 (6%)
Belamaf ongoing	4 (11%)

* One patient was in CR prior to belamaf start; ** Patient had asecretory disease and was switched to another therapy after two cycles of belamaf, due to severe keratopathy before response evaluation could be performed. *** PD was counted as the primary reason for belamaf termination, although patients who were discontinued due to PD might have also had adverse events; **** One patient died in a car accident, one patient was discontinued because worsening of general condition.

**Table 3:** Adverse events.

Characteristic:	Total Cohort *n* = 36 (100%)
Degree of Keratopathy described (KVA-Scale): No (%)	
0	9 (25%)
1	6 (17%)
2	9 (25%)
3	5 (14%)
4	7 (19%)
Time from belamaf start to first documented visual acuity impairment in days: Median (range)	41 (18–96)
Belamaf cycles until keratopathy: Median (range)	2 (1–3)
Thrombocytopenia (CTCAE): No (%)	
0	17 (47%)
1	11 (31%)
2	0
3	4 (11%)
4	4 (11%)
Infections (CTCAE): No (%) *	
0	28 (78%)
1	2 (6%)
2	2 (6%)
3	4 (11%)
4	0
Hospitalization ≥1 time: No (%)	8 (22%) **
Other AE	6 (17%) ***

* *n* = 6 with respiratory tract infections (*n* = 3 SARS-CoV-2; *n* = 1 Streptococcus pneumoniae; *n* = 2 no bacteria/virus detected); *n* = 2 with urinary tract infections; ** *n* = 5 with infections, 1 patient after accident unrelated to belamaf, 1 patient for radiation, 1 patient due to thrombocytopenia with daily Thrombocyte transfusion *** *n* = 2 liver toxicity (hyperbilirubinemia); *n* = 2 CKD Grad 3; *n* = 1 diarrhea; *n* = 1 muscular cramps.

**Table 4:** Belamaf—Literature overview.

	DREAMM-1 PMID: 30442502	DREAMM-2 PMID: 31859245	DREAMM-3 PMID: 37793771	DREAMM-6 PMID: 39433730	ALGONQUIN trial PMID: 38177852	Hultcrantz et al. [25] PMCID: PMC10429714	Alegre et al. [26] PMID: 36509945	Abeykoon et. al. [27] PMID: 35694818	Shragai et al. [28] PMID: 36205375	Our Data
Design	Prospective	Prospective	Prospective	Prospective	Prospective	Retrospective	Retrospective	Retrospective	Retrospective	Retrospective
Protocol	3.4 mg/kg Belamaf Single Agent	Cohort 1: 2.5 mg/kg Single Agent	Cohort 1: Belamaf 2.5 mg Single Agent	4 Cohorts * (1.9 mg/kg and 2.5 mg/kg in different intervals + Lenalidomide + Dexamethasone)	*** Belamaf (Dose: 1.9 mg; 2.5 mg or 3.4 mg /kg; Q4 W or Q8 W) + Pomalidomide + Dexamethasone	n.a.	2.5 mg/kg Single Agent	2.5 mg/kg (no further information)	2.5 mg/kg and 3.4 mg/kg initial dose	2.5 mg/kg and 1.9 mg/kg; single agent (69%) and combination therapies (31%)
n	35 (Part 2)	95 (Cohort1)	218 (Belamaf Cohort)	45 (all patients)	87 (all patients)	184	33	38	106	36
Age	60 (46–75)	65 (60–70)	68 (IQR: 59–74)	68 (36–80)	67 (36–35)	69 (n.a.)	70 (46–79)	67 (49–90)	69	66 (54–87)
No of prior therapy lines	n.a. ≥5 lines (57%)	7 (3–21)	4 IQR (3–4)	3 (1–10)	3 (1–6)	n.a. 62% ≥5 prior ty lines	5 (3–8)	8 (2–15)	6 (2–11)	4 (2–9)
ORR (≥PR)	60%	31%	41%	67%	88%	74%	42%	29%	46%	64%
Median PFS	7.9 months	2.9 months	11.2 months	18.4 months	21.8 months	4.5 months	3 months	2 months	4.7 months (8.8 if ≥PR)	7.3 months
Median OS	not yet sufficiently mature	not yet sufficiently mature	21 months	n.a.	34 months	7.9 months	13	7.2 months	14.5 months	20.1 months
Keratopathy/ ocular toxicity any grade	63%	71%	12% **	78% (Keratopathy)	71%	41% (Keratopathy)	52% (Keratopathy)	69% (Keratopathy)	68% (Keratopathy)	75%
Keratopathy/ ocular toxicity Grade 3–4	9%	27%	4%	n.a. “Ocular AE Grade 3–4: 69%”	55%	n.a.	21%	14%	41%	33%
Time to onset of keratopathy	23 (1–84)	36 (19–143)	n.a.	n.a.	n.a.	39 (n.a.)	n.a.	42	n.a.	41 (18–96)
Time to resolution of keratopathy	35 (5–442)	71 (57–99)	n.a.	n.a.	n.a.	n.a.	n.a.	72 (15–126)	n.a.	76 (36–380)
Thrombocytopenia any grade	57%	35%	34%	53%	44%	n.a.	21%	n.a.	27%	53%
Thrombocytopenia Grade 3–4	34%	20%	22%	29%	33%	n.a.	n.a.	n.a.	18%	22%

* A: 1.9 mg/kg Stretch; B: 1.9 mg/kg Single; C: 2.5 mg/kg Split; 2.5 mg/kg Single) ** Study investigators were not required to report pure corneal exam findings as AEs, so the incidence of keratopathy does not reflect the totality of cases that occurred during the study. Corneal exam findings were captured in the database and used to assess the KVA grade. *** Phase 1/2-Study: Dose escalation *n* = 61; recommended part 2 dose (2.5 mg/kg Q8W) *n* = 38.

## Data Availability

The data presented in this study are available on request from the corresponding author. The data are not publicly available due to ethical and privacy restrictions related to patient confidentiality. Please contact Lina Rüsing [lina.ruesing@meduniwien.ac.at] for data sharing inquiries.

## Supplementary Materials

The following supporting information can be downloaded at: https://www.mdpi.com/article/10.3390/cancers17142398/s1, Figure S1: Time to first observed belamaf response; Figure S2: Time from belamaf stop until first observed ophthalmologic improvement; Figure S3: Number of belamaf cycles; Table S1: Patient characteristics in Responder vs. Non-Responder. *p* Values were given if *p* ≤ 0.3; Table S2: 1.9 mg/kg vs. 2.5 mg/kg; *p* Values were given if *p* ≤ 0.3.

## Abbreviations

The following abbreviations are used in this manuscript:

AEs	Adverse Events
ADC	Antibody–Drug Conjugate
BCMA	B-cell Maturation Antigen
BCVA	Best-Corrected Visual Acuity
CR	Complete Response
EK	Ethikkommission (German: Ethics Committee)
IMiDs	Immunomodulatory Drugs
IMWG	International Myeloma Working Group
KVA	Keratopathy and Visual Acuity (Scale)
MM	Multiple Myeloma
mAb	Monoclonal Antibody
MECs	Microcyst-like Epithelial Changes
MMAF	Monomethyl Auristatin F
MoAbs	Monoclonal Antibodies
NCI	National Cancer Institute
ORR	Overall Response Rate
OS	Overall Survival
PD	Progressive Disease
PFS	Progression-Free Survival
PIs	Proteasome Inhibitors
PR	Partial Response
RRMM	Relapsed/Refractory Multiple Myeloma
VGPR	Very Good Partial Response
χ^2^	Chi-Square Test
