# Supplementary material for: Optimizing Belantamab Mafodotin in Relapsed or Refractory Multiple Myeloma: Impact of Dose Modifications on Adverse Events and Hematologic Response in a Real-World Retrospective Study
Source: Cancers (Basel). 2025 Jul 19;17(14):2398. doi: 10.3390/cancers17142398 (PMC12294064; doi:10.3390/cancers17142398)
Supplement: Supplementary file 1 [file cancers-17-02398-s001.zip › cancers-3746404-supplementary.pdf]

Supplementary data

Supplemental Table S1

		RESPONSE (≥PR) n=23	NO RESPONSE n=12	p-Value
**Patient:**	Age: Median (range)	66 (55-83)	68 (55-86)	
	Sex – Women: No (%)	14 (61%)	7 (58%)	
	Time since diagnosis in years: Median (range)	3.9 (1.2-12.3)	4.7 (1.6-21.7)	
**Disease:**	MM type: No (%)			
	intact	12 (52%)	10 (83%)	
	FLC	10 (43%)	2 (17%)	
	Asecretory (no Paraprotein kown)	1 (4%)	0	
	HR Cytogenetic markers No (%)			
	High risk	8 (35%)	3 (25%)	
	Standard risk	6 (26%)	3 (25%)	
	No Info	9 (39%)	6 (50%)	
	EMD: No (%)	3 (13%)	4 (33%)	0.15
	Bone-related Plasmocytoma: No (%)	6 (26%)	4 (33%)	
	Osteolytic lesions: No (%)	19 (39%)	12 (100%)	0.17
**Prior therapies:**	No of prior therapy lines: Median (range)	**4.5 (3-9)**	**3 (2-6)**	**0.015**
	Best prior observed response to any line: no (%)			
	Complete Response	15 (65%)	8 (66%)	
	Previous PIs: No (%)			
	≥2	20 (87%)	11 (92%)	
	Previous Imids: No (%)	17 (74%)	9 (75%)	
	≥2			
	Prior PACE: No (%)	3 (13%)	3 (25%)	
	Prior ASCT: No (%)	16 (70%)	7 (58%)	
	Complete response to most recent tx line: No (%)	4 (17%)	4 (33%)	0.29
	PFS to last tx line in months: Median (range)	11.4 (0.5-37)	4.7 (0.5-21.7)	0.3
**Laboratory values:**	dFLC in mg/l: Median (range)	408 (7.6-4514)	409 (3-4512)	
	Beta-2-Microglobulin in mg/l: Median (range)	3.7 (1.5-11.8)	3.3 (2.2-7.8)	0.27
	LDH in U/L: Median (range)	178 (90-2037)	212 (128-821)	
	M-Protein in g/l: Median (range)	5.7 (0.7-42)	8 (1.9-41)	0.092
**Belamaf:**	Therapy regimen used: No (%)			
	Blenrep Mono	15 (65%)	9 (75%)	
	Blenrep in Combination with other therapies	8 (35%)	3 (25%)	
	Therapy interval ≥4weeks	12 (52%)	2 (17%)	0.07
	Treatment dose: No (%)			
	2.5mg/kg (all cycles)	6 (26%)	4 (33%)	
	1.9mg/kg (≥1cycle)	17 (74%)	8 (67%)	
	1.9mg/kg (all cycles)	9 (39%)	5 (41%)	

***Table S1 Patient characteristics in Responder vs. Non-Responder**. P Values were given if p≤0.3.*

Supplemental Table S2

		1.9mg/kg n=15	2.5mg/kg n=21	p-Value
**Patient:**	Age: Median (range)	71 (56-87)	65 (54-82)	0.069
	Sex – Women: No (%)	8 (53%)	14 (67%)	
	Time since diagnosis in years: Median (range)	4.3 (1.2-11.4)	4.4 (1.6-12.3)	0.28
**Disease:**	MM type: No (%)			
	intact	11 (73%)	12 (57%)	
	FLC	4 (27%)	8 (38%)	
	Asecretory	0	1 (5%)	
	HR Cytogenetic markers No (%)			
	High risk	4 (27%)	7 (33%)	
	Standard risk	4 (27%)	5 (24%)	
	No Info	7 (47%)	9 (43%)	
	EMD: No (%)	3 (20%)	4 (19%)	
	Bone-related Plasmocytoma: No (%)	4 (27%)	7 (33%)	
	Osteolytic lesions: No (%)	13 (87%)	19 (90%)	
**Prior therapies:**	No of prior therapy lines: Median (range)	3 (2-7)	4 (2-9)	
	Best prior observed response to any line: no (%) Complete Response	10 (67%)	14 (67%)	
	Previous PIs: No (%)			
	≥2	14 (93%)	18 (86%)	
	Previous Imids: No (%)	12 (80%)	15 (71%)	
	≥2			
	Prior PACE: No (%)	1 (7%)	5 (23%)	0.19
	Prior ASCT: No (%)	7 (47%)	16 (76%)	0.069
	Complete response to most recent tx line: No (%)	4 (27%)	4 (19%)	
	PFS to last tx line in months: Median (range)	**17 (1.6-36.9)**	**4.1 (0.5-24.2)**	**0.021**
**Laboratory values:**	dFLC in mg/l: Median (range)	323 (7.7-4514)	472 (2.8-4510)	0.2
	Beta-2-Microglobulin in mg/l: Median (range)	3.6 (1.5-18.7)	3.5 (2.1-11.8)	
	LDH in U/L: Median (range)	184 (101-821)	177 (90-2037)	0.2
	M-Protein in g/l: Median (range)	5.7 (3.4 -19)	7.5 (0.7-42)	
**Belamaf:**	Therapy regimen used: No (%)			
	Blenrep Mono	13 (87%)	12 (57%)	
	Blenrep in Combination with other therapies	2 (13%)	9 (43%)	0,077
	Treatment dose: No (%)			
	2.5mg/kg (all cycles)	0	10 (48%)	
	1.9mg/kg (≥1cycle);)	0	11 (52%)	
	1.9mg/kg (all cycles)	15 (100%)	0	
	Treatment dose: No (%)			
	2.5mg/kg (all cycles)	0	10 (48%)	
	1.9mg/kg (≥1cycle)	0	11 (52%)	
	1.9mg/kg (all cycles)	15 (100%)	0	
	Belamaf cycles used. Median (range)	4 (1-11)	5 (1-27)	
	Dosing interval in days: Median (range)	33 (21-58)	30 (21-80)	
**Adverse events:**	Keratopathy (any grade)	12 (80%)	15 (71%)	
	Keratopathy (≥Grade 3)	**1 (7%)**	**11 (52%)**	**0.004**
	**Thrombocytopenia (any grade)**	**5 (33%)**	**14 (67%)**	**0.048**
	Thrombocytopenia (≥Grade 4)	1 (7%)	7 (33%)	0.064
	Infections (any grade)	2 (13%)	6 (29%)	0.25
	Infections (≥Grade 3)	2 13%)	2 (10%)	

Table S2: 1.9mg/kg vs 2.5mg/kg; *P Values were given if p≤0.3.*

Supplemental Figure S1

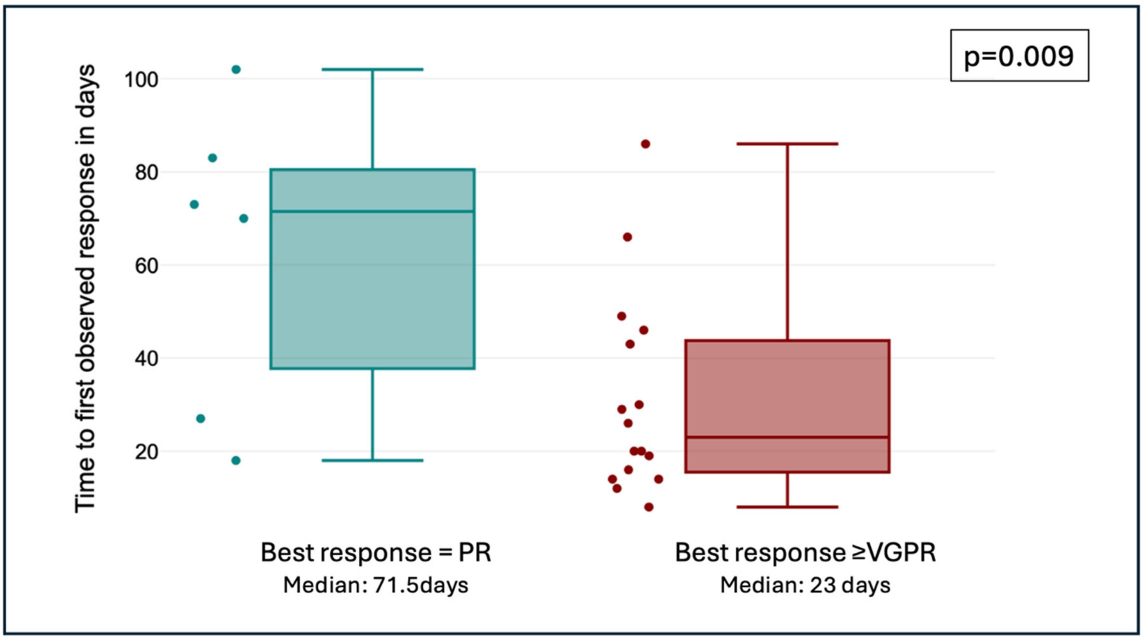

Supplemental Figure S1: Time to first observed belamaf response: Comparison between Patients with PR as best observed response and ≥VGPR

Supplemental Figure S2

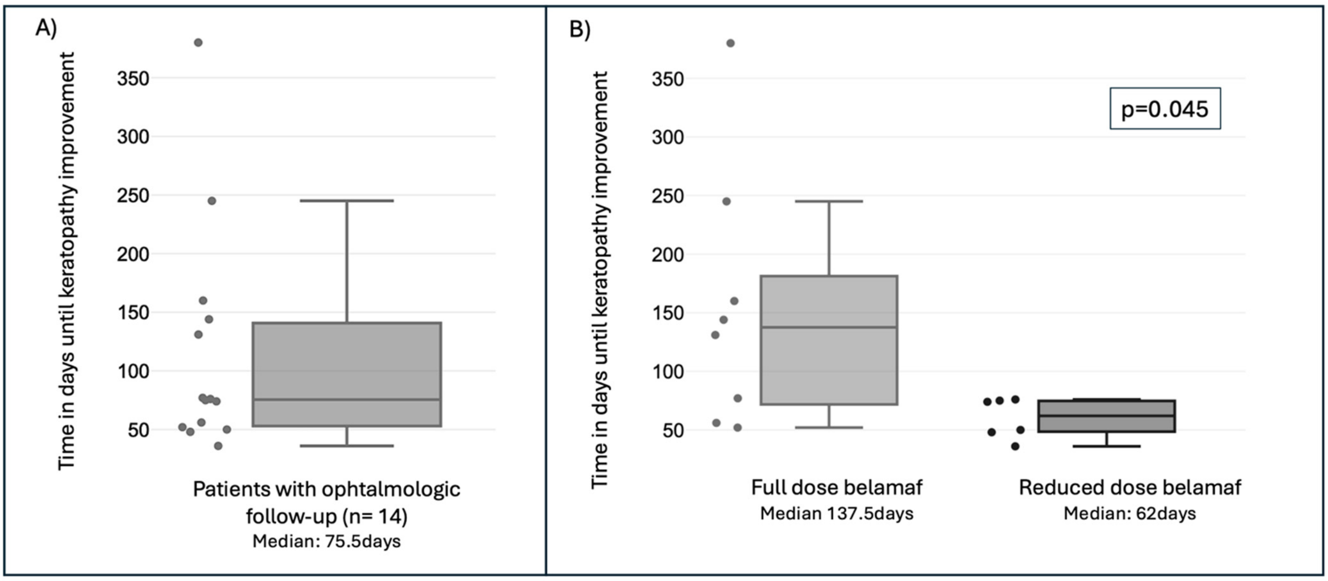

Supplemental Figure S2: Time from belamaf stop until first observed ophthalmologic improvement. A) All (n=14) patients with ophthalmologic follow-up; B) Comparison full dose cohort and reduced dose cohort.

Supplemental Figure S3

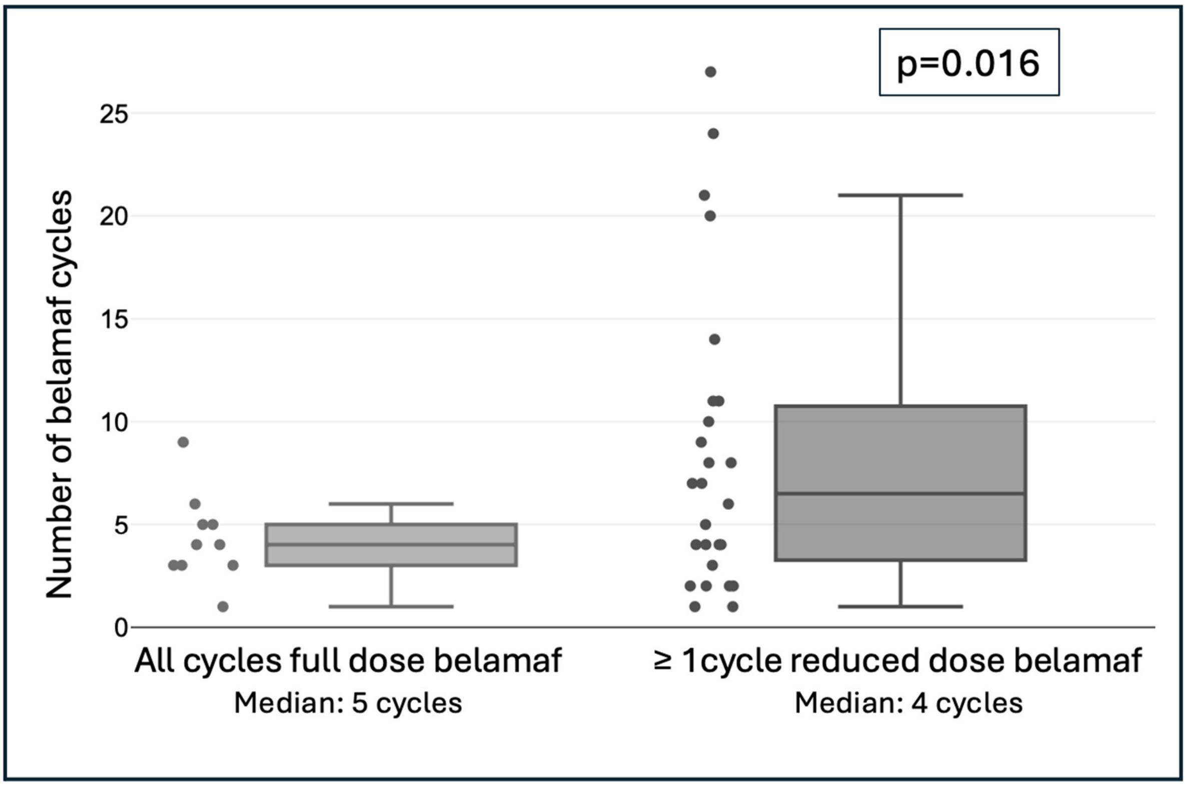

Supplemental Figure S3: Number of belamaf cycles; Comparison: all cycles in full dose and ≥ 1 cycle in reduced dose
